# UVA Irradiation Enhances Brusatol-Mediated Inhibition of Melanoma Growth by Downregulation of the Nrf2-Mediated Antioxidant Response

**DOI:** 10.1155/2018/9742154

**Published:** 2018-02-18

**Authors:** Mei Wang, Guangwei Shi, Chunxiang Bian, Muhammad Farrukh Nisar, Yingying Guo, Yan Wu, Wei Li, Xiao Huang, Xuemei Jiang, Jörg W. Bartsch, Ping Ji, Julia Li Zhong

**Affiliations:** ^1^Key Laboratory of Biorheological Science and Technology, Ministry of Education, Chongqing University, Chongqing 400044, China; ^2^Stomatological Hospital of Chongqing Medical University, Chongqing Municipal Key Laboratory of Oral Biomedical Engineering of Higher Education, Chongqing Key Laboratory of Oral Diseases and Biomedical Sciences, Chongqing 401147, China; ^3^Interdisciplinary Research Center in Biomedical Materials (IRCBM), COMSATS Institute of Information Technology, Lahore 54000, Pakistan; ^4^Department of Neurosurgery, Philipps-University Marburg, Baldingerstrasse, 35033 Marburg, Germany

## Abstract

Brusatol (BR) is a potent inhibitor of Nrf2, a transcription factor that is highly expressed in cancer tissues and confers chemoresistance. UVA-generated reactive oxygen species (ROS) can damage both normal and cancer cells and may be of potential use in phototherapy. In order to provide an alternative method to treat the aggressive melanoma, we sought to investigate whether low-dose UVA with BR is more effective in eliminating melanoma cells than the respective single treatments. We found that BR combined with UVA led to inhibition of A375 melanoma cell proliferation by cell cycle arrest in the G1 phase and triggers cell apoptosis. Furthermore, inhibition of Nrf2 expression attenuated colony formation and tumor development from A375 cells in heterotopic mouse models. In addition, cotreatment of UVA and BR partially suppressed Nrf2 and its downstream target genes such as HO-1 along with the PI3K/AKT pathway. We propose that cotreatment increased ROS-induced cell cycle arrest and cellular apoptosis and inhibits melanoma growth by regulating the AKT-Nrf2 pathway in A375 cells which offers a possible therapeutic intervention strategy for the treatment of human melanoma.

## 1. Introduction

Malignant melanoma (MM) is one of the most prevalent cancers in the Western world and is a highly aggressive dermatological malignancy associated with poor patient prognosis. The majority of MM arise from congenital melanocytic nevi or are due to a family history of MM; however, in some cases, 50% MM can also be associated with repeated intermittent sporadic ultraviolet (UV) exposure [1, 2], mostly UVB radiation plays a dominant role in the development of malignant melanoma, but the role of UVA is still unclear and controversial [3].

The progressive accumulation of genetic and environmental alterations causes disruption of homeostatic pathways, resulting in tumor cell invasion and lymphatic or haematogenous dissemination to distant sites [4]. In addition, B-Raf gene mutations are activated in 70% of human malignant melanomas [4, 5]. Over the past decades, the incidence of malignant melanoma is steadily rising [6]. Although significant advances have been made in diagnosis and treatment of MM, therapy resistance and metastasis are still the major reasons for mortality of patients [7]. Recently, some reports showed that Nrf2 expression in melanoma is related to invasion thereby worsening melanoma-specific survival [8]. Furthermore, aberrant activation of Nrf2 has been shown to be involved in chemoresistance and radioresistance of various malignant tumors, such as glioma and gastric cancer [9–11]. Thus, it is highly desirable to investigate novel therapeutic strategies capable to enhance the efficacy of metastatic melanoma treatments with fewer side effects. Nrf2 suppression and subsequent low-dose UVA irradiation might be a potential auxiliary regimen for melanoma (low dose of UVA has no carcinogenesis).

Nuclear factor E2-related factor 2 (Nrf2), a transcription factor belonging to the cap‘n'collar family of leucine-zipper (b-ZIP) proteins, has been reported to play an essential role in regulation of the cellular defense against chemicals and oxidative stress [12, 13]. However, Nrf2 is highly expressed in many cancer tissues, thereby increasing an unwanted resistance against chemotherapy, and might activate cell proliferation and suppress apoptosis [14, 15]. In addition, Nrf2 is activated by numerous oncogenic signaling pathways such as the PI3K/protein kinase B (Akt) pathway [16].

Under oxidative stress conditions including chemicals, UV irradiation, and heat shock, Nrf2 binding to its upstream keap1 (Kelch-like erythroid cell-derived protein with CNC homology- (ECH-) associated protein 1) is disrupted and leads to Nrf2 nuclear translocation and consequently activates expression of cytoprotective genes such as heme oxygenase 1 (HO-1), NAD(P)H:quinone oxidoreductase-1 (NQO1), and glutathione S-transferase (GST) drug transporters to dissipate redox homoeostasis [17, 18]. Stable activation of Nrf2 increased the resistance of human breast adenocarcinoma and neuroblastoma against tert-butylhydroquinone (tBHQ) [19]. Conversely, suppression of the Nrf2-mediated antioxidant defense system sensitizes cancer cell to ionizing radiation and chemotherapeutic drugs [17, 20, 21]. Furthermore, Nrf2 knockout mice significantly enhance the sensitivity to acetaminophen hepatotoxicity [22], cisplatin-induced nephrotoxicity [23], and bleomycin-induced pulmonary injury and fibrosis [24]. Since Nrf2 hampers cancer cell treatment, it has been analyzed as a promising drug target to combat chemoresistance [14, 19] and, up to now, a few effective Nrf2 inhibitors have been reported [25].

BR is a quassinoid isolated from *Brucea javanica* plant and has extensive pharmacological activities such as antimalarial, anti-inflammatory, and ant-tumor activity [26], primarily due to induction of proliferation arrest and activation of cell differentiation [27–29]. Recently, it was reported that BR is a potent inhibitor of Nrf2 activation thereby leading ultimately to tumor growth inhibition and ameliorated chemoresistance as in case of cisplatin [30–33]. We have found that RNA interference of Nrf2 in human skin fibroblasts increases long wave UVA- (320–400 nm) mediated damage [34], while Hirota et al. showed that Nrf2^−/−^ 3T3 mouse fibroblasts exert increased UVA-mediated apoptosis and necrosis [35].

Medium and high doses of UVA irradiation cause oxidative stress, penetrate deeply into the dermis and subcutaneous layer [36, 37], and mediate oxidative damage to biomolecules such as proteins, lipids, carbohydrates, and nucleic acids (DNA and RNA) through reactive oxygen species (ROS) triggered by endogenous photosensitization [38].

UVA exposure following 4-thiothymidine treatment markedly increased cancer cell death [39], and reactive oxidative stress inhibits distant metastasis of human melanoma cells [40]. Thus, UVA-mediated oxidative stress offers a potential source for a novel photochemotherapy. Since BR is a specific inhibitor of Nrf2, downregulation of its expression may potentiate the therapeutic effect of phototherapy in combination with an Nrf2-inhibiting drug such as BR. We therefore speculated that cotreatment of UVA radiation and BR may have synergistic effects in the treatment of melanoma.

Using BR and low-dose UVA irradiation in A375 melanoma cells, we found that cotreatment (UVA + BR) inhibited melanoma cell growth and proliferation both *in vitro* and *in vivo* and induces cell apoptosis. Suppression of Nrf2 expression causes further accumulation of cellular ROS following UVA irradiation, which in turn inhibits AKT signaling. Our experiments revealed that cotreatment of UVA and BR caused an inhibition of AKT-Nrf2 cascades and reduced melanoma growth. Thus, this cotreatment can be a novel therapeutic attempt to enhance the effectiveness of melanoma treatment with less or no side effect compared to existing treatment options.

## 2. Materials and Methods

### 2.1. Chemicals and Reagents

Dulbecco's Modified Eagle Medium (DMEM) high glucose, DMEM without phenol red, and RPMI 1640 medium were obtained from Life Technologies (Gibco, USA). Fetal calf serum was purchased from Biological Industries (BI, Israel). Penicillin and streptomycin were obtained from North China Pharmaceutical Co. Ltd. (NCPC, China). Dimethyl sulfoxide (DMSO) and 4,6-diamidino-2-phenylindole dihydrochloride (DAPI) were obtained from Sigma. CellTiter 96 Aqueous One Solution Cell Proliferation Assay was obtained from Promega (USA). Nrf2 and NQO1 primary antibodies were purchased from Santa Cruz Biotechnology Inc. (USA). HO-1, GSTP, Bcl-2, Bcl-xL, Bax, I*κ*B*α*, COX2, caspase-3, caspase-7, caspase-8, caspase-9, and PARP primary antibodies were obtained from Cell Signaling Technology (CST, USA). *β*-Actin antibody was purchased from Beijing Zhongshan-Golden Bridge Biological Technology (China). Annexin V-FITC Apoptosis Detection Kit was obtained from Beyotime Institute of Biotechnology (China). BR was purchased from Dingchen Technology (China).

### 2.2. Irradiation of Cells with UVA

UV light therapy system (Lifotronic) 365 nm (peak) spectrum lamp was used to irradiate cells (in PBS) following standard procedures, while nonirradiated cells were used as a background control (control = 0 kJ/m^2^). Following UVA irradiation, cells were incubated in conditional medium for the required time.

### 2.3. Cell Lines and Cell Culture

Human malignant melanoma A375 cell line was bought from Shanghai Cell Resource Center (Chinese Academy of Sciences, China). HaCaT cells were kindly provided by Dr. Rex M. Tyrrell (University of Bath, UK). Cells were cultured in DMEM (Gibco) high glucose or RPMI 1640 (Gibco) supplemented with 10% FBS (Biological Industries), 100 U/ml penicillin, and 150 U/ml streptomycin. Cell cultures were incubated in a humidified cell incubation chamber adjusted at 37°C with 5% CO_2_. BR was dissolved in DMSO and further diluted by triple distilled sterilized water, so that the DMSO content in cell culture medium was not higher than 3%.

### 2.4. Cell Viability Assay

Cell viability was measured by the 3-(4,5-dimethylthiazol-2-yl)-5-(3-carboxymethoxyphenyl)-2-(4-sulfophenyl)-2H-tetrazolium, inner salt (MTS, 3582, Promega), which monitors cell growth in response to treatment at certain time points. A375 cells (3000 per well) were seeded into 96-well plates and incubated overnight. Cells were then treated with various concentrations of BR and UVA alone or in combinations for times indicated. After that, cells were washed twice with PBS and 100 *μ*L MTS prepared in 10% DMEM was added and incubated for 2 hours to read the OD values at 490 nm.

### 2.5. Western Blot Assay

A375 cells were cultured for 24–30 h and treated with either UVA or BR or both for the indicated times. After specific time points, treated/nontreated cells were lysed in RIPA lysis buffer (Beyotime, P0013B). To detect the phosphorylation status of various proteins, cell lysates were prepared and extracted with SDS lysis buffer (Beyotime, P0013G), in the presence of 1 mM phenylmethylsulfonyl fluoride (Beyotime, ST506). Protein concentrations were measured by using the BCA protein assay kit (Beyotime, P0010), and 50 *μ*g protein per lane was separated by SDS-PAGE and transferred to polyvinylidene difluoride membranes (PVDF). After transfer, membranes were blocked with 5% nonfat milk in TBST for 1 hour at 37°C and incubated with the respective primary antibodies overnight at 4°C. Membranes were washed three times with 1X-TBST and incubated with horseradish peroxidase-conjugated secondary antibodies. The signals were recorded by ECL reagent (Thermo Scientific) and visualized by VersaDoc imaging system (Bio-Rad, USA).

### 2.6. Immunofluorescence Staining with FITC and Costaining with DAPI

Cells cultured in 24-well plates were washed three times with PBS and fixed with 4% formaldehyde for 20 minutes, followed by permeabilization using 0.1% Triton X-100 in PBS for 10 minutes. After washing with PBS, cells were blocked with 1% BSA 20 minutes at room temperature. The following operations were carried out in the dark: to each well, 200 *μ*l FITC (diluted to 1 : 30) was added and incubated for 1 hour at room temperature. After washing with PBS three times, 1 mg/ml DAPI (in 200 *μ*l) was incubated for 20 minutes. After washing with PBS twice, cells were mounted and analyzed under a fluorescence microscope.

### 2.7. Flow Cytometry and Cell Cycle Analysis

A375 cells were treated with BR and UVA, and apoptotic cells were detected using an Annexin V-FITC Apoptosis Detection Kit (Beyotime), followed by flow cytometry. For cell cycle analysis, A375 cells were treated with either BR or UVA or both, fixed with 70% ethanol, incubated with propidium iodide (PI) and RNase A mixture, and analyzed by flow cytometry (Becton Dickinson, USA).

### 2.8. Xenograft Assay in Nude Mice

Athymic nude (*nu/nu*) mice were obtained from Chongqing Medical University. 4-week-old male mice were injected with A375 cells (3 × 10^6^ cells) in the right flanks into the subdermal space. Tumor volumes were estimated every other day by caliper measurements, and tumor volumes were calculated by the formula (volume = tumor length in mm × width^2^ in mm × 0.5236). Once tumors reached a mean volume of 30–50 mm^3^, mice were randomly allocated into four groups and treated with either DMSO or BR (2 mg/kg) or UVA (75 kJ/m^2^) and in combination of BR and UVA every other day for seven days. Mice were sacrificed, and tumors were dissected. Formalin-fixed, paraffin-embedded tumor tissue sections were used for IHC, whereas snap-frozen tissues were subjected to Western blot analysis.

### 2.9. qRT-PCR

Incubation of A375 cells was performed with UVA, BR, or both for times indicated. Treated cells were lysed with TRIzol (Takara) for total RNA purification. Reverse transcription was performed using Go Script™ Reverse Transcription System (Promega). The qPCR analysis was performed by using a RT^2^ SYBR Green/Fluorescein PCR Master Mix (Promega) on an iQ5 real-time PCR system (Bio-Rad) with oligonucleotide primer pairs to detect various genes. All samples were normalized to GAPDH mRNA levels, and relative mRNA expressions were analyzed using 2−^△△Ct^ method as described previously [41].

### 2.10. Colony Formation Assay

A375 cells were plated at densities of 500 cells per well in 6-well plates. After 24 hours of incubation, cells were exposed to UVA (75 kJ/m^2^) and/or BR (50 nM) and the colonies formed were photographed after one week. Colonies were confirmed only if a single clone contained more than 50 cells. Fresh DMEM (10% FBS) was replaced every 72 hours.

### 2.11. Statistical Analysis

All experiments were performed three times in independent experiments to obtain reproducible results. Statistical data were subjected to analysis of variance (ANOVA) following Tukey's test to analyze the differences. A *P* value of <0.05 was considered as statistically significant.

## 3. Results

### 3.1. Low-Dose UVA Modulates the Expression of Phase II Detoxification Enzyme

**Figure 1:**
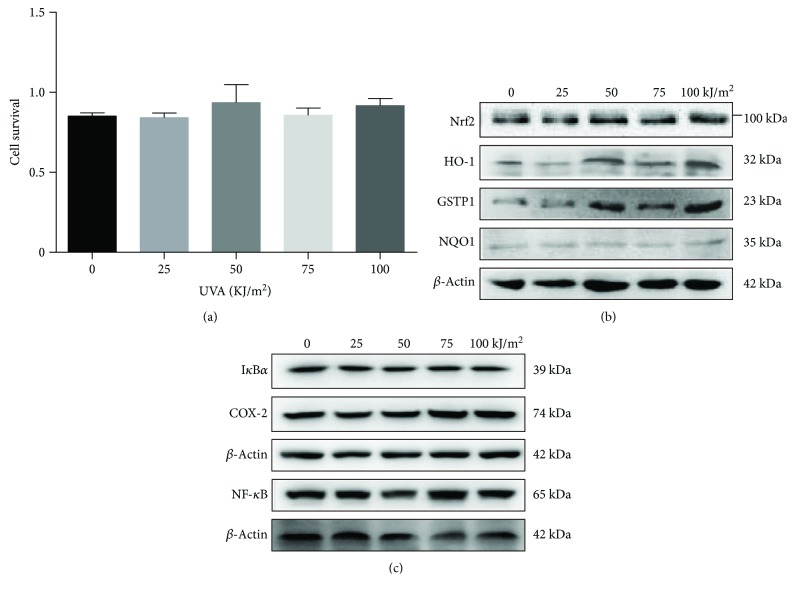
Low-dose UVA can modulate the expression of phase II detoxification enzymes. (a) A375 cells were plated overnight and irradiated with different doses of UVA, and cell survival was determined 24 hours after UVA irradiation. (b and c) Cells were treated with UVA in the range of 0–100 kJ/m^2^ for 24 hours. Total cell extracts were prepared for 24 hours following UVA irradiation and subjected to Western blot using antibodies for Nrf2, HO-1, NQO1, GSTP1, I*κ*B*α*, COX2, NF-*κ*B, and actin. We received a similar result in three independent experiments.

To explore the effect of UVA on growth of melanoma cells, A375 melanoma cells were treated with different doses of UVA. Low dose of UVA irradiation did not significantly affect survival of cells at UVA doses of up to 100 kJ/m^2^ (Figure 1(a)). Western blot results showed that these doses of UVA irradiation induced the expression of Nrf2, HO-1, and GSTP1 proteins, with slight induction of NQO1 protein (Figure 1(b)). We chose the low dose of 75 kJ/m^2^ for the following experiments since cell survival at this dose was not affected but caused an increase in the protein levels of HO-1 and a slight increase of the GSTP1 and NQO1 (Figure 1(b)).

Nrf2 and NF-*κ*B are transcription factors. It was previously reported that Nrf2 and NF-*κ*B simultaneously accumulate in the cell nucleus and NF-*κ*B (p65) antagonizes Nrf2-induced gene transcription [42]. Conversely, some phase II-inducers (enzyme) activate the signaling and inhibit NF-*κ*B pathway [43]. To understand the relationship between Nrf2 and NF-*κ*B in our experimental setup, we analyzed the NF-*κ*B signaling pathway by Western blot assay. NF-*κ*B and COX2 expression were slightly increased following UVA irradiation in a dose-dependent manner (Figure 1(c)). However, the levels of I*κ*B*α* as NF-*κ*B target proteins were not affected by UVA irradiation (Figure 1(c)), indicating that this low-dose UVA irradiation does not significantly affect the NF-*κ*B signaling pathway.

### 3.2. BR Potentially Inhibits the Nrf2 Signaling Pathway

**Figure 2:**
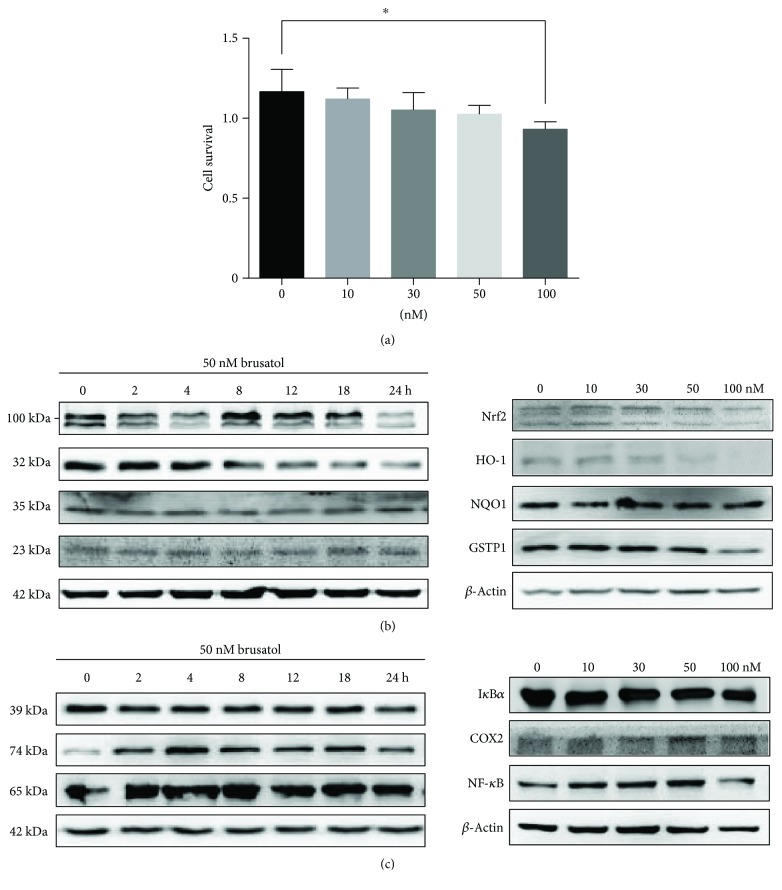
BR specifically inhibits the Nrf2 signaling pathway. A375 cells were treated with BR for 24 hours. (a) Cell survival was assessed using an MTS assay. (b and c) Cells were treated with various concentrations of BR for 24 hours ((b), right; (c) right) or with 50 nM BR for different time interval as indicated ((b), left; (c) left). Total cell extracts were prepared and subjected to Western blot using antibodies for Nrf2, HO-1, NQO1, GSTP1, I*κ*B*α*, COX2, NF-*κ*B, and actin. Data in (a) are shown as mean ± SD; ^∗^ *P* < 0.032. *P* values are based on control versus treatment.

First, we examined the effect of BR on cell viability. A375 cells were treated with a concentration range of BR (0–100 nM), and the proliferation rates of A375 cell were slightly reduced in a dose-dependent manner (Figure 2(a)). To confirm that BR inhibits the Nrf2 pathway in A375 cells, whole-cell lysates were collected and protein expression levels were determined by Western blotting after treatment with BR. After 24 hours, BR caused a reduction of Nrf2 and GSTP1 protein levels in a concentration-dependent manner, whereas the levels of NQO1 remained relatively unchanged (Figure 2(b), right).

A time course of BR (50 nM) treatment of A375 cells showed an effect on the Nrf2 pathway and revealed that Nrf2 protein levels were significantly decreased at 2, 4, and 24 hours following the addition of BR, with maximal reduction at 4 hours (Figure 2(b), left). Significant changes in downstream targets of Nrf2, that is, HO-1 and NQO1, were not observed over the time course, whereas only a slight reduction in GSTP1 was observed (Figure 2(b), left). Further, we did not find that 50 nM BR had an effect on NF-*κ*B and I*κ*B*α*, whereas the COX2 protein levels were slightly increased compared to controls (Figure 2(c)). A previous study demonstrated that BR induces activation of NF-*κ*B in HL-60 cell [44]. This difference in NF-*κ*B activation is most likely due to a cell line-specific effect, and a concentration of 50 nM BR is required for this effect. These results suggest that the predominant effect of BR in A375 cells is a potential inhibition of the Nrf2 signaling pathway.

### 3.3. Cotreatment Increases Intracellular ROS Level and Inhibits A375 Cell Growth and Proliferation

**Figure 3:**
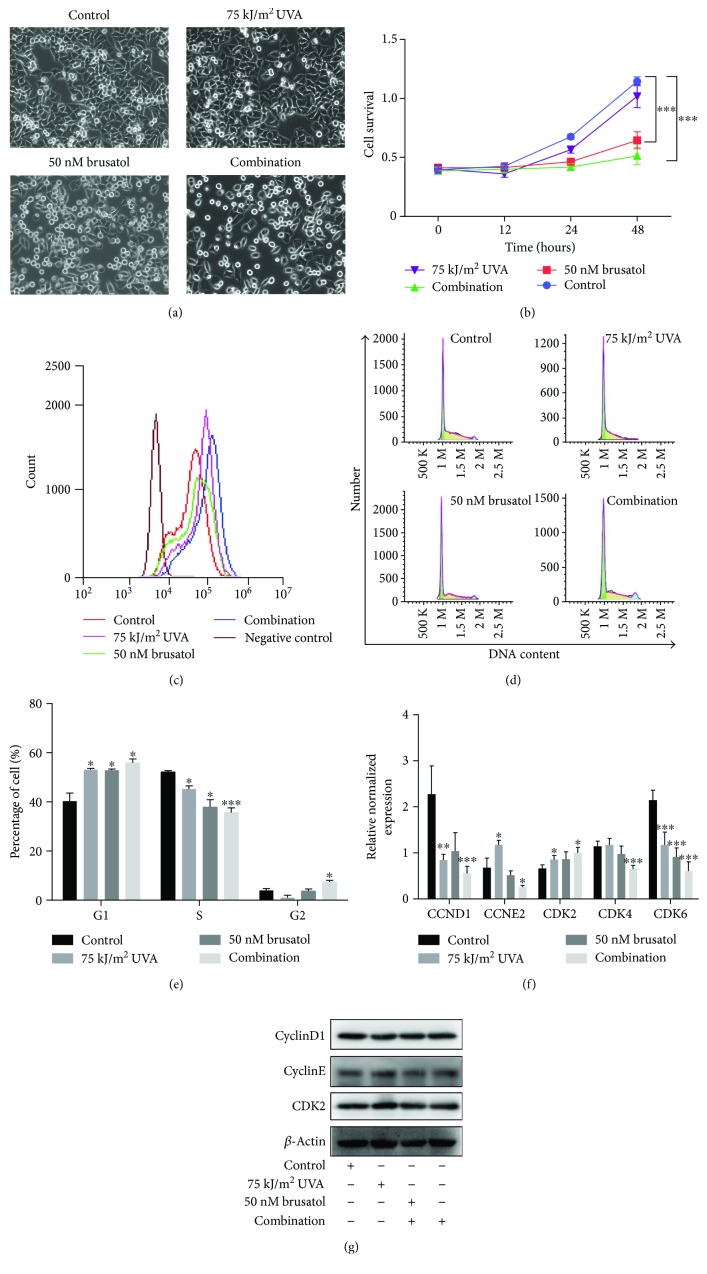
Cotreatment increases intracellular ROS level and inhibits A375 cell growth and proliferation. (a) A375 cells were treated with UVA and BR for 24 hours. Cell morphology was observed, and cell survival analyses were performed by an MTS assay (b). Cell cycle analysis by PI staining (d and e) and cell cycle-related genes cyclinD1, cyclinE2, CDK2, CDK4, and CDK6 were performed by qRT-PCR assay (f). (c) A375 cells were pretreated with BR for 2 hours prior to UVA exposure and were then irradiated with low-dose UVA (75 kJ/m^2^). ROS levels were determined by flow cytometry immediately following UVA irradiation. All data are shown as the mean ± SD; ^∗^ *P* < 0.05, ^∗∗^ *P* < 0.01, and ^∗∗∗^ *P* < 0.001. All *P* values are based on analysis control versus treatment.

We hypothesize that a relatively low-dose UVA irradiation of 75 kJ/m^2^ combined with 50 nM BR can affect cell survival in A375 cells. Therefore, we addressed the question whether cotreatment with UVA and BR resulted in lower cell survival in conjunction with elevated intracellular ROS levels and hence an increased sensitivity to BR treatment. Intracellular ROS levels were measured in A375 cells using flow cytometry. Low-dose UVA-treated A375 cells displayed slightly increased ROS levels compared to nontreated cells. Likewise, BR treatment increased intracellular ROS levels slightly, so that cotreatment further increased ROS levels (Figure 3(c)). To determine the efficacy of BR and UVA on cell growth, A375 cells were treated with BR or UVA and cell proliferation were counted by MTS. As shown in Figures 3(a) and 3(b), combination treatment significantly suppressed cell proliferation. We also check the proliferation marker Ki67 in BR- or UVA-treated cells, and the results were similar as that for cell survival (Supplementary Figure 1).

Furthermore, the cell cycle of A375 cells was analyzed by flow cytometry to examine whether cotreatment inhibits cell proliferation by inducing a cell cycle arrest. UVA treatment plus BR resulted in a marked increase in the percentage of A375 cells in the G1 phase (Figures 3(d) and 3(e)). To confirm these results, we analyzed the mRNA levels of cyclinD1, cyclinE2, CDK2, CDK4, and CDK6 which could promote A375 cells to pass the G1 to S phase checkpoint. We found that cyclinD1, cyclinE2, CDK4, and CDK6 were reduced following UVA/BR cotreatment, while CDK2 expression was increased (Figure 3(f)) at gene expression level, but this gene expression was different in translational level. Immunoblot assays revealed that cylinE2 and CDK2 were slightly decreased following UVA/BR cotreatment, but no significant change was seen in cyclinD1 expression (Figure 3(g)). Collectively, these results suggest that UVA is able to enhance BR-induced ROS levels and affects cell survival and the regulation of cell cycle-related proteins.

### 3.4. Cotreatment Blocks Nrf2 and AKT Signaling through Enhanced Cell Apoptosis

**Figure 4:**
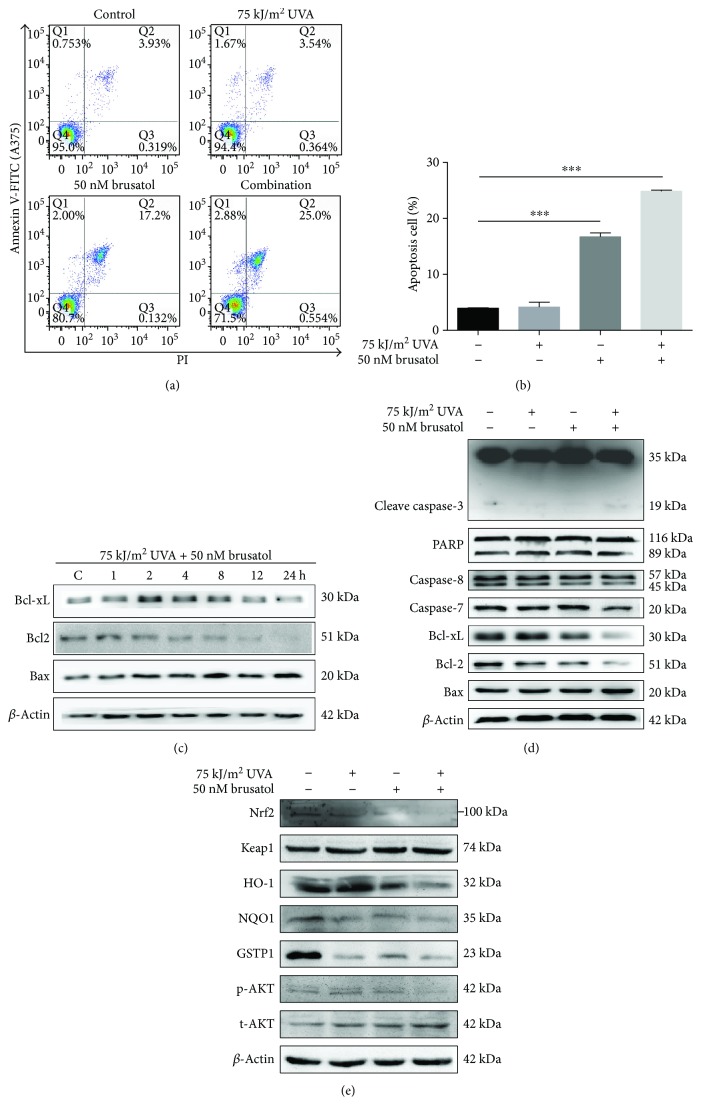
Cotreatment inhibits Nrf2 and AKT pathways and leads to apoptosis in A375 cells. (a and b) A375 cells were treated with UVA and BR for 24 hours before measuring apoptosis using Annexin V and PI in conjunction with flow cytometry. (c and d) Whole-cell lysates from A375 cells were prepared and subjected to immunoblotting using antibodies against Bcl-2 family proteins, PARP, cleaved caspase-3 (C-caspase3), caspase7, caspase8, and actin. Cells were treated with UVA, BR, or both for 24 hours or different time intervals as indicated. (e) Immunoblot analysis using antibody against Nrf2 pathway proteins, AKT and p-AKT protein from A375 cell lysates. ^∗^ *P* < 0.05, ^∗∗^ *P* < 0.01, and ^∗∗∗^ *P* < 0.001. All *P* values are based on analysis control versus treatment.

To explore whether the observed reduced cell viability of A375 cells was due to apoptosis, A375 melanoma cells and HaCaT skin keratinocytes were stained with Annexin V-FITC and PI and analyzed by flow cytometry. The results showed that HaCaT cells do not exert significant apoptosis after treatment with either BR alone or cotreatment under this condition (Supplementary Figure 2). In contrast, BR or cotreatment in A375 cells leads to significant apoptosis detected 24 hours post treatment, with a higher apoptosis rate in A375 cells after cotreatment (Figures 4(a) and 4(b)). Furthermore, UVA/BR cotreatment markedly suppressed protein levels of Bcl-2 and Bcl-xl and increased the expression levels of Bax. Consistent with these findings, UVA/BR cotreatment causes cleavage and activation of caspase-3, clearly indicating a marked increase in apoptosis rate (Figures 4(c) and 4(d)).

To evaluate the effect of cotreatment on the Nrf2 pathway in the presence of UVA, we performed immunoblot assays to detect protein levels of Nrf2 and its downstream genes. The expression levels of Nrf2, HO-1, and GSTP1 were decreased 24 hours following cotreatment compared with UVA or BR single treatment (Figure 4(e)). In addition, we found that AKT phosphorylation at position Ser473 was markedly reduced 24 hours after application of UVA/BR, whereas total AKT was not significantly changed (Figure 4(e)). These results indicate that cotreatment in A375 inhibits/AKT signaling while inhibiting Nrf2 expression. Similar results were obtained with camptothecin as another potential inhibitor of Nrf2 (data not shown) [45, 46].

### 3.5. Combination Treatment Inhibited Tumor Growth in Mice

**Figure 5:**
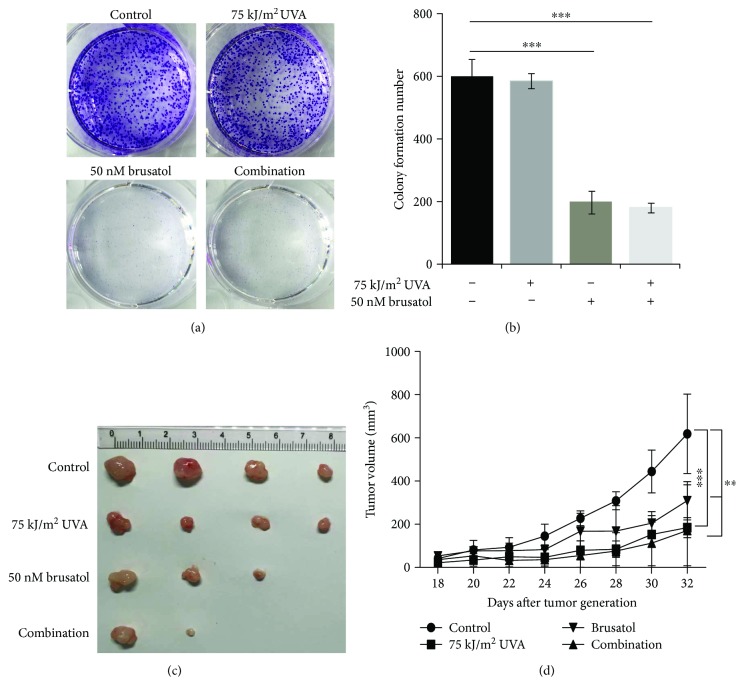
Cotreatment causes reduction of melanoma cell-derived tumors *in vivo*. (a and b) A375 cells were treated with UVA and BR for 7 days. At days 2, 4, and 6, UVA and BR treatment was repeated. After 7 days, colony formation was examined by staining the resulting colonies with crystal violet. Colonies with more than 50 cells were counted (*n* = 3). (c) A375 cells (3.0 × 10^6^) were injected subcutaneously into the right flank of 4-week-old male nude mice (*n* = 4 for each group). When tumor size reached 30~50 mm^3^, intraperitoneal injections of BR (2 mg/kg) or irradiation with UVA (75 kJ/m^2^) or with both were administered every other day for five times. (d) Xenograft tumor sizes were measured after mice bearing tumors were treated with either vehicle (control), UVA alone, BR (2 mg/kg), or with a combination of UVA and BR for 10 days (*n* = 4). All data are shown as the mean ± SD; ^∗∗^ *P* < 0.01 and ^∗∗∗^ *P* < 0.001. All *P* values are based on the analysis of control tumors versus treated ones.

Since the observed apoptosis induction by combined treatment with UVA/BR occurs in melanoma cells, but not in skin keratinocytes (HaCaT cells) under these conditions (data not show), we addressed the question whether a combination of UVA and BR can affect cell proliferation in melanoma-derived tumors *in vitro* and *in vivo* (Figure 5). For assessment of tumor growth in vitro, a colony formation assay was performed. Both BR alone and in combination with UVA reduced the number of colonies formed, while UVA has no significant effect on colony formation (Figures 5(a) and 5(b)). To explore the effect of UVA/BR cotreatment on tumor growth *in vivo*, A375 xenografts were grown in NOD/SCID mice as a heterotopic tumor model. NOD/SCID mice were injected with A375 cells (3 × 10^6^ per injection site) to form a tumor. After 18 days, tumors reached volumes of about 30 to 50 mm^3^ and UVA (75 KJ/m^2^) irradiation or BR (2 mg/kg) was administered intraperitoneally every other day for 10 days [47]. Mice were covered with silver paper, contact and irradiate the tumors using a UVA lamp, and tumor growth was observed for 5 days after cessation of treatment. We observed a significant inhibition of tumor growth *in vivo* after cotreatment (Figures 5(c) and 5(d)) that was significantly higher than after single treatments of the tumors. Moreover, in one out of four cases treated with BR alone, a complete remission of the tumor and in two cases after UVA/BR cotreatment was observed. These observations demonstrate that cotreatment of melanoma-derived tumors reduced the tumor growth *in vivo*.

### 3.6. UVA Enhances Nrf2 Knockout-Mediated Cell Suppression

**Figure 6:**
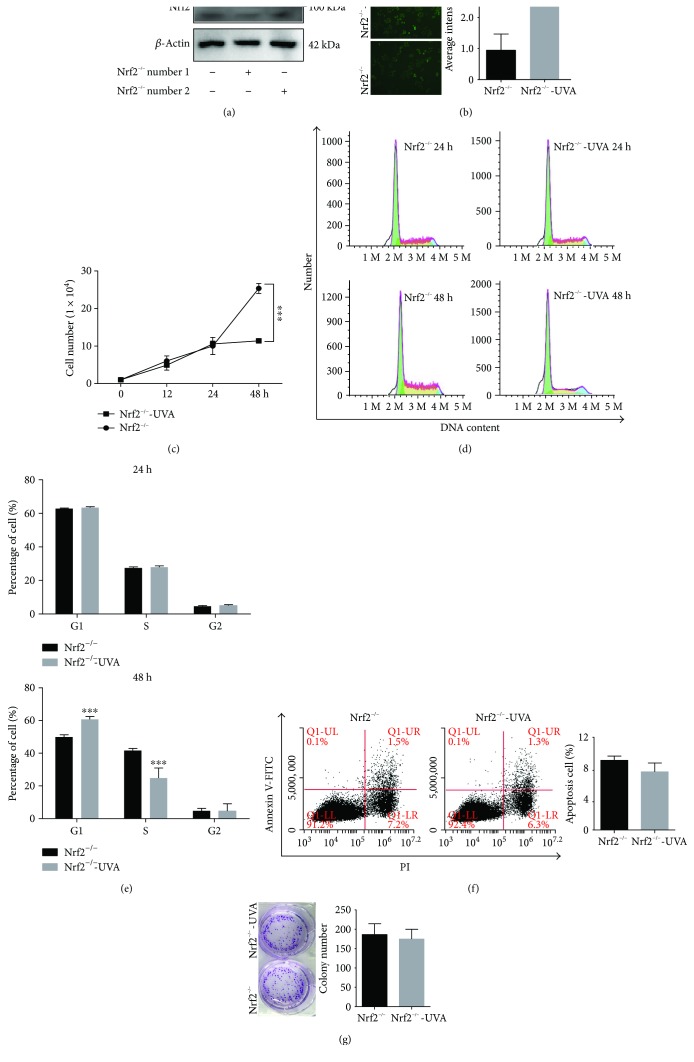
UVA irradiation leads to suppression of A375 Nrf2 knockout cells. (a) Western blot assay was used to analyze protein expression levels of Nrf2 in A375 cells. (b) DCFH-DA-based ROS quantification. (c) A375 Nrf2^−/−^ irradiated with 75 kJ/m^2^ UVA, followed by MTS assay (d) cell cycle analysis by PI staining after 24 and 48 hours. (f) Apoptosis assay using Annexin V and PI to analyzed Nrf2^−/−^ cells after 48 hours irradiation. (g) Colony formation was examined by staining colonies with crystal violet. Colonies with more than 50 cells were counted (*n* = 3). ^∗∗∗^ *P* < 0.001.

In addition to the pharmacological inhibition of Nrf2 using BR, we examined the effect of a genetic knockout of Nrf2 in A375 cells using the CRISPR/Cas9 method. Cas9 plasmids that expressed Nrf2 gRNA sequences against Nrf2 were used, and the clone number 1 identified bearing the knockdown, and Nrf2^−/−^ clone number 2 exhibited the most significant reduction of Nrf2 protein expression (Figure 6(a)). Therefore, subsequent experiments were performed with this cell clone named Nrf2^−/−^ while Cas9 clones of A375 cells were used as a control cell line for the knockout cell line. First, we determined whether UVA enhances intracellular ROS in A375 Nrf2 knockout cells leading to increased sensitivity against UVA treatment. Furthermore, we investigated intracellular ROS levels in Nrf2^−/−^ cells following UVA irradiation and observed increased ROS levels compared to Nrf2^−/−^ cells (Figure 6(b)).

To determine the efficacy of UVA on cell growth in Nrf2^−/−^ cells, cells were treated with UVA (75 kJ/m^2^) and cell proliferation was quantified by MTS (Figure 6(c)). UVA irradiation suppressed proliferation of Nrf2^−/−^ cells at 48 hours when compared to sham control, and the result of Nrf2^−/−^ number 1 similar to Nrf2^−/−^ number 2 (Supplementary Figures 3A and 3C). Furthermore, flow cytometry was used to examine whether UVA irradiation inhibits proliferation of Nrf2^−/−^ cells by inducing cell cycle arrest. Representative histograms and combined results are summarized (Figures 6(d) and 6(e)). UVA treatment resulted in a marked increase of the percentage of G1 phase in Nrf2^−/−^ cells at 48 hours (Figures 6(d) and 6(e)). To explore whether the reduced cell viability was due to apoptosis, Nrf2^−/−^ cells were irradiated with UVA and flow cytometer analysis revealed no increase in apoptosis in Nrf2^−/−^ cells (Figure 6(f)). To further assess the effect of UVA irradiation on Nrf2^−/−^ cell proliferation, a colony formation assay was conducted. UVA irradiation has no effect on colony formation in Nrf2^−/−^ cells (Figure 6(g)), and we also get the similar results in Nrf2^−/−^ number 1 cell (Supplementary Figure 3B). These results demonstrate that this dose of UVA mediates cell suppression, but not apoptosis in Nrf2^−/−^ cells. Previous studies suggested that 150 kJ/m^2^ UVA irradiation induced cell death in Nrf2-deficient murine dermal fibroblasts [33]. The different results may due to a difference in UVA dose (75 kJ/m^2^) applied in our study and to the variable UVA response in different cell lines.

## 4. Discussion

Nrf2 is a key player of the cellular defense against endogenous and exogenous chemical and oxidative insults. Exposure to chemicals may cause organ damage, so that the lung, liver, and kidney are significantly more affected in Nrf2 knockout mice than in their latter controls [48], hence made them highly prone to develop oxidative damage-related diseases and cancers. Moreover, Nrf2 has been proposed as an effective target for cancer chemoprevention and chemoresistance due to its linkage to pathways such as NF-*κ*B pathway [49]. Recently, some studies reported that BR provokes a rapid and transient inhibition of Nrf2 signaling and sensitizes hepatoma cells to chemical toxicity [13]. As demonstrated, increased levels of Nrf2 contribute to resistance in therapies (radio- and chemotherapy) in breast and lung cancer [49]; on the contrary, inhibition of Nrf2 may lead to enhanced efficacy of photo- or chemotherapy. In this study, we have shown that UVA enhances BR chemosensitivity in an additive fashion and that cotreatment inhibits cell proliferation and induces apoptosis *in vitro* and ameliorates melanoma growth *in vivo*.

BR is a broad spectrum anti-inflammatory agent that stabilizes lysosomal membranes, thereby reducing the release of hydrolytic enzymes that cause damage to surrounding tissues [50]. The anticancer properties of BR were demonstrated in lymphocytic leukemia, Ehrlich carcinoma, and hepatoma [51–53] and are mainly due to a significant inhibition of chemoresistance-mediating Nrf2 gene and the resulting downstream target genes, thereby sensitizing tumor cells to chemo- and phototherapy [30, 31, 37]. Previously, it was reported that low concentrations of BR may inhibit general protein synthesis, but more recently, it was shown that BR acts as a Nrf2 pathway-specific inhibitor when used in an upper nanomolar range [19, 48, 53]. Here, we also demonstrated that BR when used in A375 melanoma cells in a range of 10–100 nM, specifically downregulated the protein level of Nrf2 and its target genes. Despite a moderate reduction of HO-1 and NQO1, we did not find a significant effect on the NF-*κ*B and its target genes.

UVA can penetrate deeply into the subcutaneous layer and primarily induces cellular responses through oxidative stress (ROS) triggered by endogenous photosensitization of the drug. UVA alone or in combination with photoreactive drugs can lead to ROS-mediated damage of biomolecules including DNA [54]. For instance, psoralen plus UVA (PUVA), a nontoxic photoreactive drug, is activated by subsequent exposure to UVA light, which causes extensive DNA damage leading to extensive tumor cell death, and clinically used to treat psoriasis as well as head and neck cancers [39, 55]. Recently, berberine has been reported to be a photosensitive drug and can be used in photodynamic therapy (PDT) to treat cancer cells, where the photosensitive drug is activated upon exposure to UVA, causing massive DNA strand breaks in tumor cells [56]. In addition, low concentration of S^4^TdR when combined with nonlethal doses of UVA kills hyperproliferative or cancerous skin cells [39].

In this study, we found that cotreatment (UVA + BR) elevated intracellular ROS and induced the Akt pathway by inhibition of Akt phosphorylation at Ser473 [57]. The Akt/PI3K pathway forms an important component of cell survival mechanisms [58, 59], and previous reports demonstrated functional interactions with Nrf2 activation [60–62]. Similarly, our results revealed that Akt phosphorylation was decreased with Nrf2 suppression in A375 cells with cotreatment.

Previously, it was demonstrated that a decrease in Nrf2 activity is associated with chemotherapeutic efficacy in mice after chronic administration of BR [30, 31]. We found that either BR alone or in a regimen with UVA and BR abolished the clonogenicity of melanoma cells. The tumor xenograft experiments in nude mice revealed that unlike cotreatment, treatment of either UVA or BR alone has an inhibitory effect on melanoma growth. However, Zhang and colleagues found that cisplatin or BR alone showed no significant inhibition of tumor growth in A549 xenografts. The difference in our results may be due to the experimental conditions or in the cell lines used.

**Figure 7:**
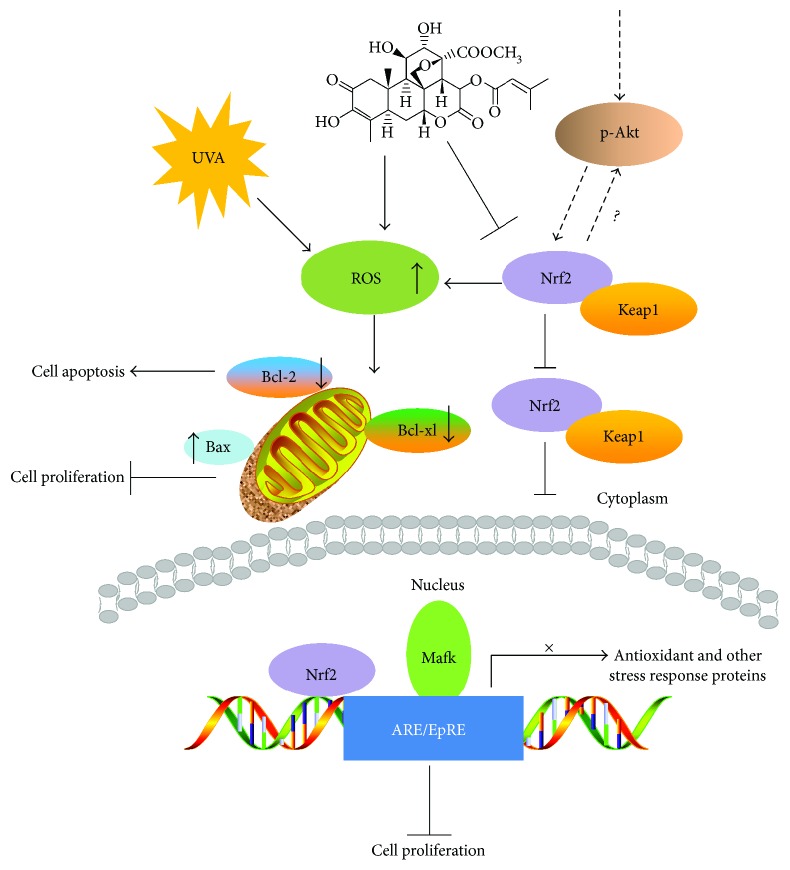
Schematic representation of combination- (UVA and brusatol) induced Nrf2 suppression in A375 cells.

We conclude that UVA/BR cotreatment inhibited melanoma cell growth and proliferation both *in vitro* and *in vivo*. Furthermore, we identified the AKT-Nrf2 pathway as mechanistically relevant for the observed antitumor effect of UVA and BR when both are combined. Therefore, we propose the usage of UVA and BR in combination as a novel treatment regimen for malignant melanomas thereby causing a prominent antitumor effect via regulation of AKT-Nrf2 pathway (summarized in Figure 7).

## Supplementary Materials

Supplementary Materials Supplementary Figure 1: cotreatment inhibited Ki67 expression. Immunofluorescence assay was used to analyze cell proliferation marker Ki67 and its expression in UVA- and BR-treated cells for 48 hours. Supplementary Figure 2: cotreatment has no effect on keratinocyte apoptosis. (A and B) Keratinocyte HaCaT cells were treated with UVA and BR for 24 hours before measuring apoptosis using Annexin V and PI in conjunction with flow cytometry. Supplementary Figure 3: UVA irradiation inhibited Nrf2^−/−^ number 1 A375 cell proliferation. (A) A375 Nrf2^−/−^ number 1 irradiated with 75 kJ/m2 UVA, followed by MTS assay. (B) Cell cycle-related genes cyclinE2, CDK4, and CDK6 were performed by qRT-PCR assay. (C) Colony formation was examined by staining colonies with crystal violet. Colonies with more than 50 cells were counted (*n* = 3).
