# Supplementary material for: UVA Irradiation Enhances Brusatol-Mediated Inhibition of Melanoma Growth by Downregulation of the Nrf2-Mediated Antioxidant Response
Source: Oxid Med Cell Longev. 2018 Feb 18;2018:9742154. doi: 10.1155/2018/9742154 (PMC5835260; doi:10.1155/2018/9742154)
Supplement: Supplementary Materials — Supplementary Figure 1: cotreatment inhibited Ki67 expression. Immunofluorescence assay was used to analyze cell proliferation marker Ki67 and its expression in UVA- and BR-treated cells for 48 hours. Supplementary Figure 2: cotreatment has no effect on keratinocyte apoptosis. (A and B) Keratinocyte HaCaT cells were treated with UVA and BR for 24 hours before measuring apoptosis using Annexin V and PI in conjunction with flow cytometry. Supplementary Figure 3: UVA irradiation inhibited Nrf2−/− number 1 A375 cell proliferation. (A) A375 Nrf2−/− number 1 irradiated with 75 kJ/m2 UVA, followed by MTS assay. (B) Cell cycle-related genes cyclinE2, CDK4, and CDK6 were performed by qRT-PCR assay. (C) Colony formation was examined by staining colonies with crystal violet. Colonies with more than 50 cells were counted (n = 3). [file 9742154.f1.pptx]

## Slide 1
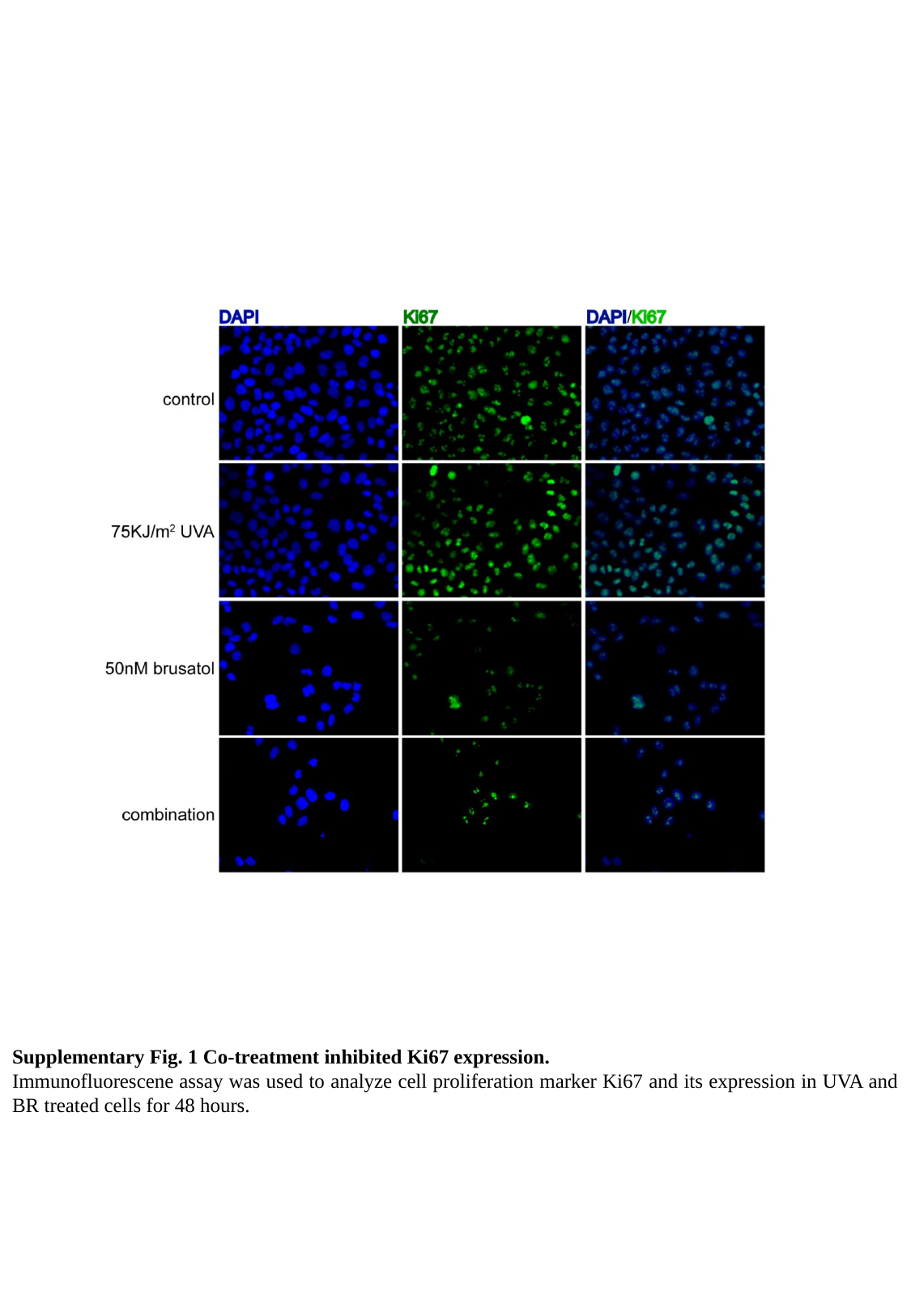

Supplementary Fig. 1 Co-treatment inhibited Ki67 expression.
Immunofluorescene assay was used to analyze cell proliferation marker Ki67 and its expression in UVA and BR treated cells for 48 hours.

## Slide 2
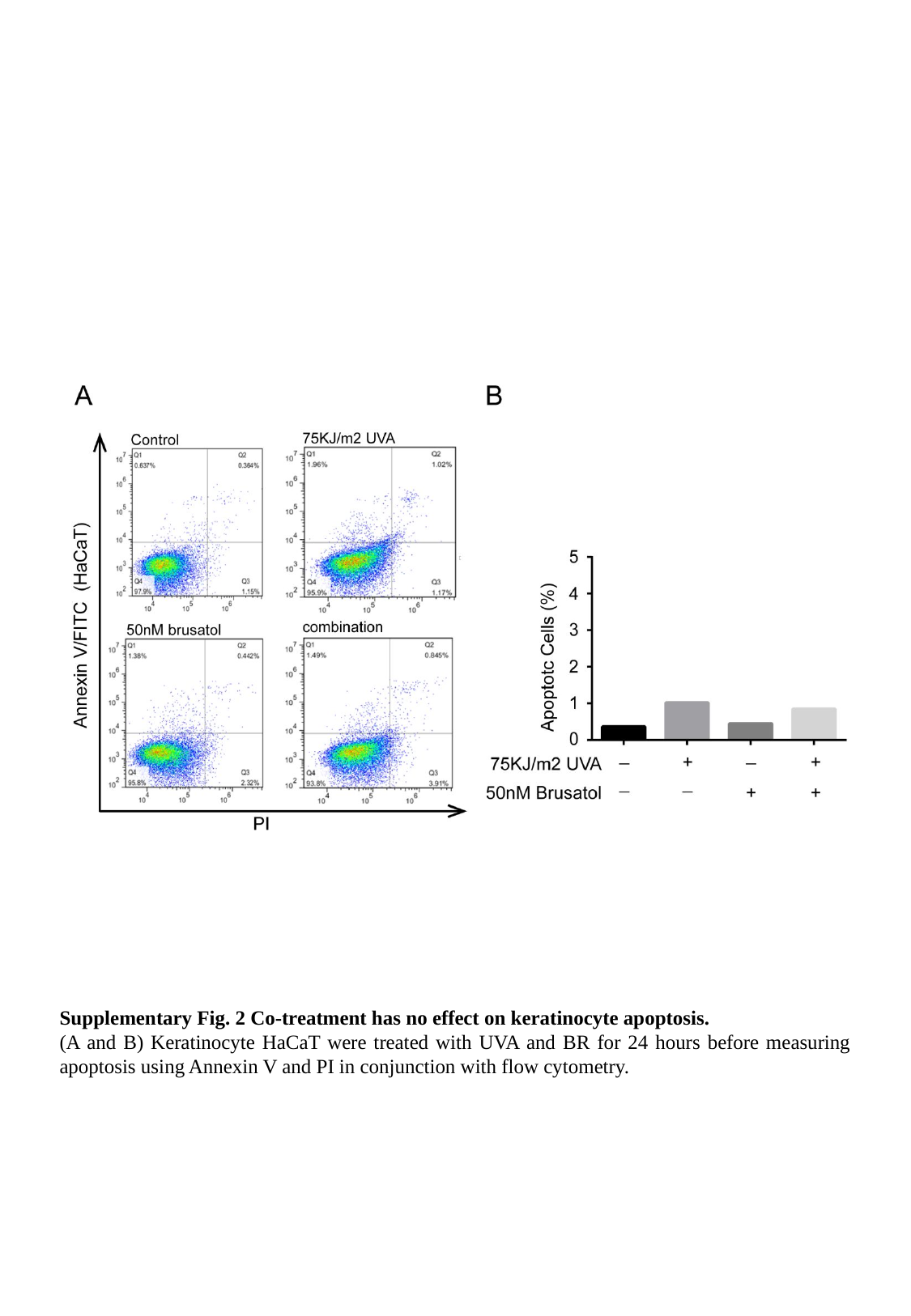

Supplementary Fig. 2 Co-treatment has no effect on keratinocyte apoptosis.
(A and B) Keratinocyte HaCaT were treated with UVA and BR for 24 hours before measuring apoptosis using Annexin V and PI in conjunction with flow cytometry.

## Slide 3
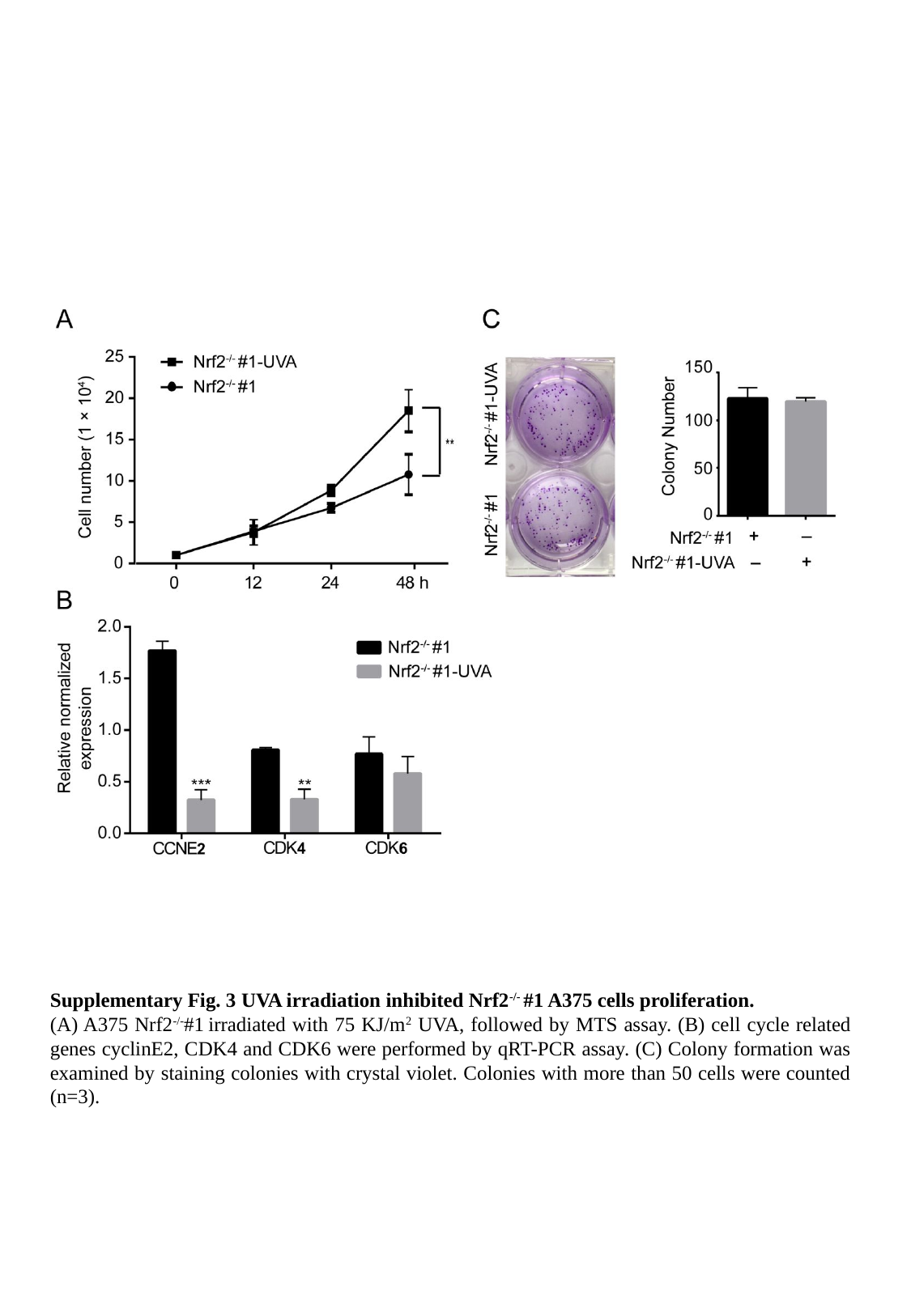

Supplementary Fig. 3 UVA irradiation inhibited Nrf2-/- #1 A375 cells proliferation.
(A) A375 Nrf2-/-#1 irradiated with 75 KJ/m2 UVA, followed by MTS assay. (B) cell cycle related genes cyclinE2, CDK4 and CDK6 were performed by qRT-PCR assay. (C) Colony formation was examined by staining colonies with crystal violet. Colonies with more than 50 cells were counted (n=3).
